# Extraterrestrial regolith is hemostatic and potentially suitable for hemorrhage control in space

**DOI:** 10.1016/j.rpth.2025.103342

**Published:** 2026-01-05

**Authors:** Nabil Ali-Mohamad, Ting-Hsuan Wang, Lih Jiin Juang, Nuoya Peng, Massimo F. Cau, Kevin Cannon, Christian J. Kastrup

**Affiliations:** 1 Michael Smith Laboratories, University of British Columbia, Vancouver, British Columbia, Canada; 2 Department of Biochemistry and Molecular Biology, University of British Columbia, Vancouver, British Columbia, Canada; 3 Versiti Blood Research Institute, Versiti Wisconsin, Milwaukee, Wisconsin, USA; 4 Departments of Surgery, Biochemistry, Biomedical Engineering, and Pharmacology and Toxicology, Medical College of Wisconsin, Milwaukee, Wisconsin, USA; 5 School of Biomedical Engineering, University of British Columbia, Vancouver, British Columbia, Canada; 6 Department of Geology and Geological Engineering, Colorado School of Mines, Golden, Colorado, USA; 7 Ethos Space Corp, Los Angeles, California, USA

**Keywords:** combat casualty care, hemostasis, *in situ* resource utilization (ISRU), intrinsic pathway, space medicine

## Abstract

**Background:**

Long-duration space missions beyond low Earth orbit pose increased risks of injury to astronauts. Traumatic hemorrhage will be a cause of preventable death. Due to payload constraints, *in situ* production of medical materials is essential. Regolith from the Moon, Mars, and asteroids is rich in silicates, which may serve as a hemostatic agent.

**Objectives:**

Here, we aimed to evaluate whether extraterrestrial regolith simulants, their mineral components, and meteorites can activate coagulation through factor (F)XII and control bleeding.

**Methods:**

The procoagulant potential of Lunar and Martian regolith simulants, their component silicate minerals, and meteorite samples was assessed *in vitro*. Plasma clotting turbidity, thrombin generation, and FXIIa chromogenic assays were performed using either normal or FXII-inhibited/immunodepleted human plasma. ζ Potential and SiO_2_ content were also plotted against time to fibrin clot formation. The *in vivo* efficacy of Lunar highland simulant (CSM-LHT-1), Mars global simulant high clay (CSM-MGS-1C), and Northwest Africa-869 chondritic meteorite was assessed in a pilot study using a porcine model of penetrating hemorrhage.

**Results:**

All extraterrestrial regolith simulant samples accelerated clotting, thrombin generation, and FXII activation in normal plasma, with reduced effects in FXII-immunodepleted or FXII-inhibited plasma. Phyllosilicates showed greater procoagulant activity than framework silicates. *In vivo*, wounds treated with regolith remained clotted longer and lost less blood than wounds treated with gauze alone, with the Northwest Africa-869 chondrite meteorite significantly improving clotting and reducing blood loss.

**Conclusion:**

Extraterrestrial regolith activated coagulation in a FXII-dependent manner and reduced blood loss in a trauma model of penetrating hemorrhage. This suggests that extraterrestrial regolith may be used as a hemostatic agent during space missions.

## Essentials

• Long-duration space missions may require locally produced products for emergency medicine.
• Lunar, Martian, and meteoric materials were assessed for their ability to stop bleeding.
• These materials enhanced clotting via FXII activation and thrombin generation in plasma.
• In a porcine trauma model, these materials reduced blood loss, supporting their use in space trauma care.

## 1 Introduction

The return of travel beyond the International Space Station and the introduction of projects that include long-term stays on the Moon or Mars carry a new set of health risks that may have previously been overlooked by astronauts. Working in larger, less controlled areas with larger equipment increases the risk of bodily injury due to penetrating, crushing, or blunt trauma [1,2]. While engineering controls and adequate designs in safety would minimize the risks of trauma in such a resource-limited setting, hemorrhage due to trauma could be a key cause of preventable death for astronauts on the frontlines of building new structures [[3], [4], [5]].

Over the last 2 decades, there have been advances in hemorrhage control technologies, along with the advent of the U.S. Department of Defense's highly specialized Tactical Combat Casualty Care guidelines for prehospital trauma care [6]. These advances may be translatable to managing unexpected and complex bleeds in the resource-limited environment of space, buying time for astronauts and preventing mortality until definitive surgical repair is possible. Recently, Combat Gauze (Z-Medica) was introduced into the first-aid packs used by astronauts aboard the International Space Station. Adequate medical equipment to manage hemorrhage must either be sent with the astronauts or possibly be prepared *in situ* prior to the start of large projects on the Moon or Mars. With other key life-saving medical equipment likely taking precedence over emergency hemostatic materials for long-term exploration missions, preparing hemostatic agents through *in situ* resource utilization (ISRU) could be lifesaving.

On Earth, many vertebrate species have evolved to express the protein coagulation factor (F)XII; in humans, FXII circulates in blood plasma at a concentration of approximately 40 mg/L [7]. FXII is present in land-dwelling mammals, which uniquely also express FXI. Following contact with select negatively charged surfaces, FXII is activated to FXIIa, which then activates FXI to FXIa, resulting in faster clot formation *ex vivo* [8]. Silicates are well-established, powerful activators of FXII. We have previously shown that FXII contributes to hemostasis in wounds contaminated with soil, a silicate-rich material [9]. Silicates form the basis of many topical hemostatic materials used for hemorrhage control [10].

This theory can be extended to consider the effects of soils from other extraterrestrial surfaces. Although chemically and mineralogically different from naturally occurring terrestrial soils, both Lunar and Martian regoliths are known to have high silicate concentrations. Anorthite-rich plagioclase is abundant in Lunar highlands regolith, while Lunar mare and Martian regolith are primarily composed of plagioclase, pyroxene, and olivine, all of which are silicate minerals [[11], [12], [13]]. S-type (silicaceous) asteroids, which are present on the surface of the Moon and Mars, account for 17% of known asteroids and are primarily composed of iron and magnesium silicates. These asteroids are thought to be the source of chondritic meteorites, which are known to have high concentrations of magnesium silicates [14]. Given these mineralogical compositions, extraterrestrial regolith should also exhibit coagulation characteristics and patterns similar to those of Earth soils.

**Figure 1:**
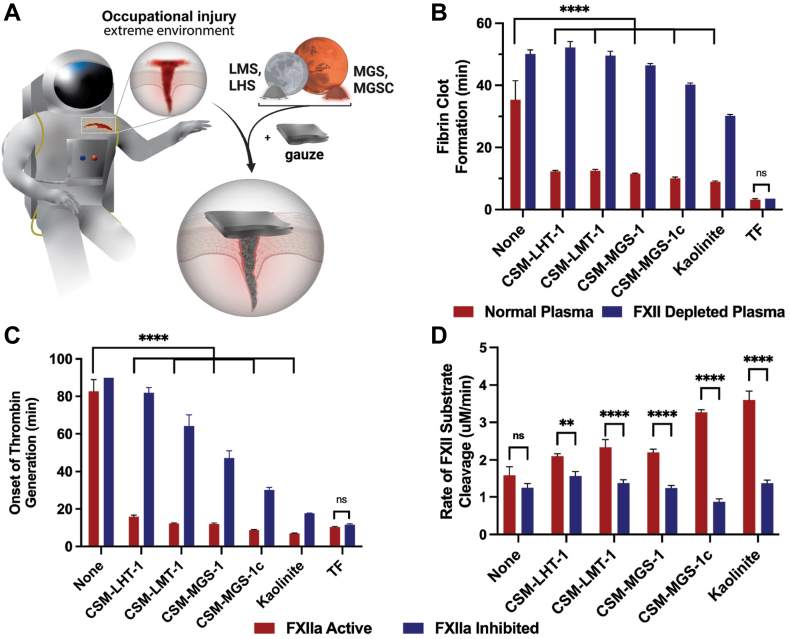
(A) Hemorrhage due to trauma from occupational injury is a potential cause of preventable death for astronauts, and regolith from the Moon (Lunar mare simulant [LMS/CSM-LMT-1] and Lunar highland simulant [LHS/CSM-LHT-1]) or Mars (Mars global simulant [MGS/CSM-MGS-1] and Mars global simulant high clay [MGSC/CSM-MGS-1c]) may be effective hemostatic and regolith simulants that accelerate clotting of plasma in a factor (F)XII-dependent manner. (B) Clotting times of normal human plasma and FXII-depleted plasma inoculated with regolith simulants and kaolinite. (C) Onset of thrombin generation in either normal human plasma (FXIIa Active) or plasma incubated with corn trypsin inhibitor (FXIIa Inhibited). (D) Rate of FXII activation by measuring cleavage of a chromogenic substrate for FXIIa. Values represent mean ± SEM. ∗∗*P* < .01, ∗∗∗∗*P* < .0001; ns, not significant (*P* > .05); analyzed by 2-way anova with Tukey’s multiple comparisons test. TF, tissue factor.

Here, we aimed to determine whether regolith simulants and chondrite meteorites could be used to trigger the coagulation cascade to halt fatal hemorrhage, given their high silicate content (Figure 1A). Although regolith simulants are analogs of actual extraterrestrial regolith, they were used due to the limited availability of the latter. However, all simulants used are well characterized and mimic the mineralogical and chemical properties of Lunar and Martian soils [13,15]. We assessed FXII activation by extraterrestrial regolith simulants (ETRS), meteorites, and their component materials. We hypothesized that ETRS and chondrite meteorites would halt hemorrhage in a porcine model of liver puncture compared with plain gauze alone.

## 2 Methods

### 2.1 Preparation of simulant regolith, silicate minerals, and meteorites

ETRS were developed by one of the authors and later commercialized by Space Resource Technologies. Meteorites were sourced from Rocks & Gems Canada. Mars global simulant (CSM-MGS-1), CSM-MGS-1 high clay (CSM-MGS-1C), Lunar highland simulant (CSM-LHT-1), Lunar mare simulant (CSM-LMT-1), and the component materials (plagioclase, pyroxene, olivine, Na-montmorillonite, Ca-montmorillonite, vermiculite, palygorskite, and serpentine) were initially ground with a mortar and pestle and sifted through a 140-mesh stainless steel sieve. Meteorite samples (Northwest Africa [NWA]-869 [ordinary chondrite], NWA-13713 [winonaite], Brenham [pallasite], Sericho/Habaswein [pallasite], Munionalusta [iron], Gibeon [iron], and Shikote-Alin [iron]) were ground to a powder with a diamond file, further crushed with a hammer, and particle sizes were selected using a 140-mesh stainless steel sieve. Si and SiO_2_ contents in the regolith were calculated from known SiO_2_ concentrations in the raw materials used to prepare the silicate mixtures that formed the simulants.

### 2.2 Plasma clotting turbidity assay

A 0.4 mg/mL suspension of the prepared simulants, meteorites, and kaolin was prepared in calcium-saline solution (40 mM CaCl_2_ and 90 mM NaCl) to recalcify and enable clotting of citrated plasma. To determine whether the ETRS activated clotting in a FXII-dependent manner, the prepared suspensions were added to either citrated normal human plasma or FXII-immunodepleted human plasma at a 1:3 volumetric ratio to yield a final ETRS concentration of 0.1 mg/mL. Results were compared with calcium-saline solution alone and with calcium-saline solution containing 30 pM tissue factor. Tissue factor was used as a positive control, as it is a strong activator of the FXII-independent extrinsic coagulation pathway. The absorbance of each sample was measured at 405 nm to assess fibrin clot formation. Clotting time was measured as half the final plateaued turbidity of the clot.

### 2.3 Thrombin generation assay in plasma

A fluorescent substrate was used to directly assess thrombin activity. The fluorescent substrate for thrombin (Boc-Asp-[OBzl]-Pro-Arg-MCA, Peptide Institute Inc) was added to the prepared ETRS suspensions, yielding a final concentration of 1 μM in the assay. Either normal human plasma or normal human plasma preincubated with corn trypsin inhibitor (CTI; Haematologic Technologies) at 75 μg/mL for 15 minutes was added to the final soil suspensions. Fluorescence was measured over 90 minutes (λ excitation/λ emission = 360/465 nm), and the onset of thrombin generation was defined as the point in time at which the slope of the fluorescence vs time graph was largest.

### 2.4 Chromogenic assay for FXIIa activity in plasma

A chromogenic assay was used to directly assess FXIIa activity. The chromogenic substrate for FXIIa (0.8 mM H-D-CHA-Gly-Arg-pNA.2AcOH, BioPacific Diagnostic Inc) was added to the prepared ETRS suspensions and to citrated normal human plasma or to normal human plasma preincubated with CTI at 75 μg/mL for 15 minutes (1:5 in 0.9% NaCl), resulting in a final ETRS concentration of 0.1 mg/mL. We note that this chromogenic substrate can also be cleaved by other plasma enzymes, including kallikrein, and that its cleavage cannot be completely attributed to FXIIa [16]. Once the substrate was added, the rate of change in absorbance was monitored immediately at 405 nm for 30 minutes at 37 °C. The rate of change in absorbance was converted to the amount of p-nitroaniline cleaved using the extinction coefficient of p-nitroaniline at 405 nm (9450 M^-1^ cm^-1^; path length = 0.28 cm).

### 2.5 ζ Potential of simulant regolith and silicate minerals

ETRS and component minerals were suspended in water at 1 mg/mL. The ζ potential of the particles was measured at 37 °C using dynamic light scattering (Zetasizer NanoZS, Malvern Panalytical).

### 2.6 Animal model and care

This study was approved by the University of British Columbia Animal Care Committee (protocol number A18-0348) and performed according to the guidelines of the Canadian Council on Animal Care. Female Yorkshire pigs (40-50 kg) received ketamine (20-30 mg/kg) and midazolam (0.1-1 mg/kg) by intramuscular injection. Animals were anesthetized with 5% isoflurane by inhalation, then promptly intubated and mechanically ventilated for the duration of the procedure. Anesthesia was maintained with isoflurane (1%-3%), propofol (2-7 mg/kg/h), and midazolam (0.4-0.7 mg/kg/h) as required. Buprenorphine (0.01-0.05 mg/kg) was administered by intramuscular injection for analgesia. Heart rate, electrocardiogram, blood pressure, peripheral capillary oxygen saturation, carbon dioxide, temperature, appearance of the skin and mucous membranes, jaw tone, and reflexes were monitored and maintained throughout anesthesia and procedures.

A laparotomy was performed, and the liver was exposed. Linear liver punctures measuring 2 cm long and 1 cm deep were created using a 20-blade scalpel to simulate a simple penetrating wound. A total of 2 g of either CSM-LHT-1, CSM-MGS-1C, or NWA-869 meteorite powder was topically applied to a 5 cm × 5 cm piece of surgical gauze and promptly manually approximated to the puncture wound. An additional surgical gauze alone was used as the control group. The gauze was removed every 2 minutes to assess hemostasis, or sooner if no oozing was observed around the approximated gauze. Each bleed was observed for up to 10 minutes from its initiation. If rebleeding occurred, the gauze was reapproximated with the same piece, and the cycle for assessing hemostasis was restarted. Blood loss was measured by lining the abdomen with preweighed gauze prior to each bleeding event and weighing after bleeding had stopped. Up to 6 individual injuries were made on each liver. The animal’s mean arterial pressure was tracked to ensure that the start of each bleeding event was within 10 mm Hg of the pig’s baseline mean arterial pressure. Primary outcomes were time spent clotted within 10 minutes and total blood loss.

### 2.7 Statistical analysis

Statistical analysis of the assay results was performed using GraphPad Prism 10.4.1. Results of the plasma turbidity assay, thrombin generation assay, and FXIIa chromogenic assay were analyzed using 2-way analysis of variance (anova) with Tukey’s multiple comparisons test. Correlation was determined by Pearson’s correlation coefficient, and a line of best fit was determined using simple linear regression. Blood loss and time to hemostasis were analyzed by the Kruskal–Wallis H test with Dunn’s multiple comparisons test. All values were considered statistically significant at *P* < .05.

## 3 Results

### 3.1 Extraterrestrial regolith accelerates clotting of human plasma by activating FXII

The hemostatic potential of 4 ETSR samples was assessed using complementary assays. In the blood plasma clotting turbidity assay, ETRS samples significantly reduced the mean time to clot formation ± SEM from 35.4 ± 6.1 minutes (*n* = 9) to 12.3 ± 0.4 minutes, 12.5 ± 0.4 minutes, 11.5 ± 0.4 minutes, and 10.1 ± 0.4 minutes for CSM-LHT-1, CSM-LMT-1, CSM-MGS-1, and CSM-MGS-1C soils, respectively, in normal blood plasma (*P* < .0001 in all cases; Figure 1B). These ETRS behaved similarly to kaolinite, a mineral well known for activating clotting [8]. In plasma immunodepleted of FXII, the presence of ETRS had a less pronounced effect on time to clot formation. FXII-depleted plasma with CSM-LHT-1, CSM-LMT-1, and CSM-MGS-1 clotted in 52.2 ± 2.0 minutes, 49.5 ± 1.5 minutes, and 46.4 ± 0.6 minutes, respectively, comparable to the 50.1 ± 1.4 minutes clotting time when calcium-saline solution was added without ETRS (*P* > .05 in all cases). However, clots formed with CSM-MGS-1C (40.2 ± 0.6 minutes) and kaolinite (30.2 ± 0.5 minutes) clotted significantly more rapidly than with calcium-saline solution alone in FXII-depleted plasma (*P* < .01 and *P* < .0001, respectively). Normal plasma treated with ETRS and kaolinite clotted significantly faster than FXII-depleted plasma (*P* < .0001 in all cases).

Time to thrombin generation is another method to assess the potential of ETRS to activate clotting. Similar to the plasma clotting turbidity assay, CSM-LHT-1 (16.0 ± 0.8 minutes), CSM-LMT-1 (12.3 ± 0.3 minutes), CSM-MGS-1 (12.1 ± 0.5 minutes), and CSM-MGS-1C (8.8 ± 0.2 minutes) generated thrombin significantly faster than the samples with calcium-saline solution alone (82.7 ± 6.3 minutes; *P* < .0001 in all cases; Figure 1C). As expected, kaolinite also significantly reduced the time to thrombin generation (7.1 ± 0.1 minutes) compared with normal calcium-saline solution (*P* < .0001). Plasma incubated with a competitive inhibitor for FXIIa (CTI) significantly increased the time to the onset of thrombin generation in all ETRS samples compared with normal plasma containing active FXIIa (*P* < .0001). However, despite the inhibition of FXIIa, and in a pattern similar to the turbidity assay, the Martian simulants still showed quicker thrombin generation times (CSM-MGS-1: 47.2 ± 3.8 minutes; CSM-MGS-1C: 30.2 ± 1.4 minutes) than the Lunar simulants (CSM-LHT-1: 81.8 ± 2.9 minutes; CSM-LMT-1: 64.2 ± 5.9 minutes), with kaolinite still being the fastest (17.7 ± 0.2 minutes). None of the FXIIa-inhibited plasma samples treated with calcium-saline solution alone showed thrombin generation over the course of the assay length (90 minutes).

To assess the direct effects of ETRS on the activation of FXII, the rate of cleavage of a FXII fluorogenic substrate was measured (Figure 1D). ETRS increased the rate of FXII substrate cleavage. While CSM-LHT-1 did not show a significantly higher rate (2.1 ± 0.1 μM/min) compared with normal buffer (1.6 ± 0.2 μM/min; *P* = .09), the other ETRS and kaolinite significantly increased FXII substrate cleavage (*P* < .05). In FXIIa-inhibited plasma, none of the ETRS or kaolinite showed an increase in substrate cleavage compared with the buffer control (range, 0.9-1.6 minutes). All the samples, except the buffer control, significantly increased the rate of FXIIa substrate cleavage in FXIIa-active plasma compared with FXIIa-inhibited plasma (*P* < .01 in all samples).

### 3.2 Identifying the mineral components of extraterrestrial regolith that accelerate clotting of human plasma through FXII

**Figure 2:**
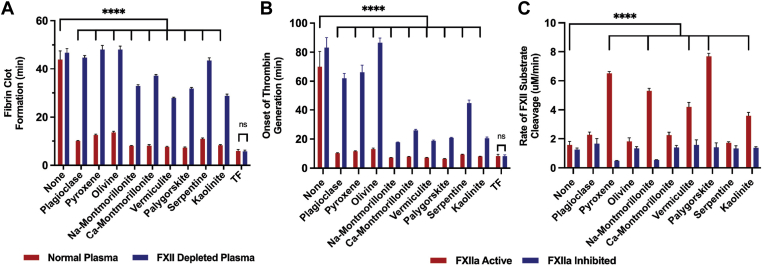
Mineral silicates, which are components of the regolith simulants, accelerate clotting of plasma in a factor (F)XII-dependent manner. (A) Time to fibrin clot formation of normal human plasma and FXII-depleted plasma inoculated with silicate minerals. (B) Onset of thrombin generation in either human plasma (FXII Active) or plasma incubated with corn trypsin inhibitor (FXIIa Inhibited). (C) Rate of cleavage of a chromogenic substrate for FXIIa. Values represent mean ± SEM. ∗∗∗∗*P* < .0001; ns, not significant (*P* > .05); analyzed by 2-way anova with Tukey’s multiple comparisons test. TF, tissue factor.

The silicate mineral components of regolith, often found in extraterrestrial environments, were also assessed separately for their hemostatic potential to further determine whether specific minerals activate FXII [13,15,17]. In these assays, plagioclase, pyroxene, olivine, Na-montmorillonite, Ca-montmorillonite, vermiculite, palygorskite, and serpentine were assessed and compared with the calcium-buffer control and kaolinite. In the plasma clotting turbidity assay (Figure 2A), all silicate minerals significantly accelerated clotting in normal plasma compared with the buffer control (*P* < .0001 in all cases). In FXII-deficient plasma, framework and chain structure silicates (plagioclase, pyroxene, and olivine), with the addition of serpentine, had a limited effect on clotting time (*P* > .05). However, the other phyllosilicates (Na-montmorillonite, Ca-montmorillonite, vermiculite, palygorskite, and kaolinite) significantly reduced the clotting time in FXII-deficient plasma. All the mineral silicates clotted normal plasma significantly faster than in FXII-deficient plasma (*P* < .0001 in all cases).

Similar trends were observed in the thrombin generation assay (Figure 2B). All the silicate minerals significantly accelerated thrombin generation in normal plasma compared with the buffer control (*P* < .0001 in all cases). In FXIIa-inhibited plasma, a more pronounced trend in the difference between the effects of framework and chain structure silicates compared with those of the other phyllosilicates was observed. Even in FXIIa-inhibited plasma, all the phyllosilicates significantly accelerated thrombin generation compared with the buffer control (*P* < .0001 in all cases). In FXIIa-inhibited plasma, olivine had no effect on thrombin generation (*P* = .99), whereas plagioclase and pyroxene accelerated the onset of thrombin generation (*P* < .001 and *P* < .01, respectively), though to a lesser extent than the phyllosilicates. All the mineral silicates significantly accelerated the onset of thrombin generation in normal plasma compared with FXIIa-inhibited plasma, though the extent of the difference was much greater for the framework and chain structure silicates (*P* < .0001) than for the other phyllosilicates (*P* range, <.0001 to <.05).

The direct effects of the silicate minerals on the activation of FXII were assessed by measuring the rate of cleavage of a FXII chromogenic substrate (Figure 2C). Substrate cleavage was considered FXIIa-like rather than definitive FXIIa activity because the substrate can also be cleaved by other plasma enzymes. In normal plasma, olivine and serpentine had no effect on FXII activation (*P* > .99), and plagioclase and Ca-montmorillonite had minimal effects on FXII activation (*P* = .32 and *P* = .38, respectively). Pyroxene and all the other phyllosilicates, however, significantly increased the rate of FXII substrate cleavage in normal plasma (*P* < .0001 in all cases). In FXIIa-inhibited plasma, none of the silicate minerals showed an increase in substrate cleavage compared with the buffer control.

### 3.3 Meteorites accelerate the clotting of human plasma

**Figure 3:**
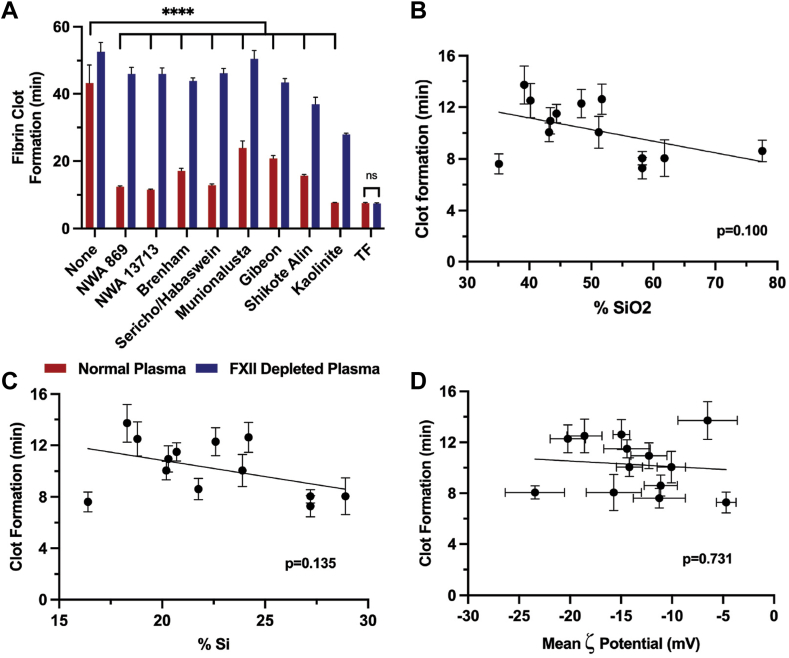
(A) Time to fibrin clot formation of normal human plasma and factor (F)XII-depleted plasma inoculated with meteorite samples. The meteorites in A have been arranged in order from highest to lowest silicate content (left to right). Kaolinite is included as a positive control. Values represent mean ± SEM. ∗∗∗∗*P* < .0001; ns, not significant (*P* > .05); analyzed by 2-way anova with Tukey’s multiple comparisons test. (B and C) Comparing the clot formation time with SiO_2_ content, (B) silicon content, (C) and ζ potential (D) of extraterrestrial regolith simulants and mineral silicates. Values represent mean ± SEM. NWA, Northwest Africa; TF, tissue factor.

A plasma clotting turbidity assay was carried out on a set of meteorite samples with known, varying silicate compositions to assess the hemostatic potential of real extraterrestrial materials (Figure 3A). All the meteorite samples significantly accelerated clotting in normal plasma compared with the buffer control (*P* < .0001 in all cases). When comparing meteorite samples with the buffer control in FXII-depleted plasma, NWA-869, NWA-13713, Sericho, and Munionalusta had minimal effects on the speed of clot formation. However, Brenham (*P* < .05), Gibeon (*P* < .05), and Shikote-Alin (*P* < .0001) significantly increased the speed of fibrin clot formation. Meteorites clotted plasma significantly slower in FXII-depleted plasma than in normal plasma in all cases (*P* < .0001).

### 3.4 Clotting time is affected by the composition of ETRS and its components

The known SiO_2_ content of the ETRS and the component silicate minerals were plotted against the average time to clot formation, as determined by the plasma clotting turbidity assay (Figure 3B). The materials had SiO_2_ concentrations ranging from 35.1% to 77.6%. Correlation analysis of this data suggested a possible relationship between SiO_2_ content and plasma clot formation, though it was not statistically significant (*P* = .10). The silicon content was also plotted against clot formation time (Figure 3C), showing a similar relationship, though not statistically significant (*P* = .14). All the samples tested in this study had negative ζ potentials, indicating a net negative charge (Figure 3D). However, in this dataset, there was limited correlation between clotting time and the magnitude of ζ potential.

### 3.5 Space regolith augments hemostasis in puncture wounds

**Figure 4:**
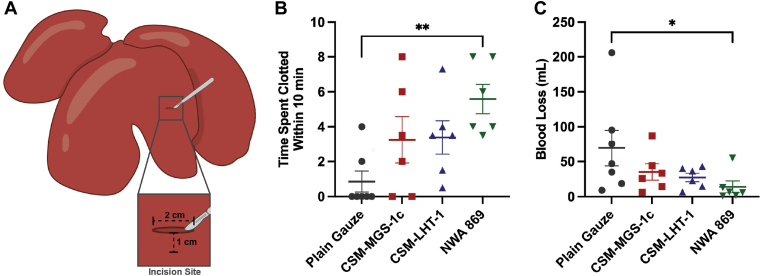
Regolith and meteorites initiate clotting and halt hemorrhage in a porcine liver injury model. (A) Schematic showing the positioning and size of puncture wounds made in the liver of pigs. (B) Time spent clotted during the 10-minute observation period for each sample tested. (C) Blood loss with plain gauze compared with extraterrestrial regolith simulants and meteorite samples. Values represent mean ± SEM. ∗*P* < .05; ∗∗*P* < .01; ns, not significant (*P* > .05); analyzed by Kruskal–Wallis H test with Dunn’s multiple comparisons test. CSM-LHT-1, Lunar highland simulant; CSM-MGS-1c, Mars global simulant high clay; NWA, Northwest Africa.

To assess the *in vivo* potential of extraterrestrial regolith to halt hemorrhage, CSM-MGS-1C, CSM-LHT-1, and NWA-869 meteorites were applied to puncture wounds made in a live porcine model of liver hemorrhage—a standard injury used to model surgical bleeding (Figure 4A) [[18], [19], [20]]. We selected CSM-LHT-1 because the Artemis Base Camp is planned for the Moon’s south polar highlands [21,22]. CSM-MGS-1C is a high-clay Martian global simulant also relevant to a potential Martian base. Selecting a site with clay-rich soils is preferred for ISRU to construct building materials and to improve soil fertility and water-extraction potential [[23], [24], [25], [26]]. NWA-869 was selected because it demonstrated strong *in vitro* hemostatic activity and is an authentic extraterrestrial material that represents meteorites found on the Moon and Mars. This model mimics surgical bleeding and is commonly used to assess hemostatic agents [19]. Four pigs were used, with 6 injuries per pig, and treatments were randomized so that each pig received 1 to 2 replicates of each treatment. Regolith powder was topically placed on a piece of plain surgical gauze and promptly applied to the puncture wound. Wounds that had only plain surgical gauze applied to them remained clotted for only 0.89 ± 0.59 minutes (*n* = 7) within 10 minutes (Figure 4B). The hemostasis time of plain surgical gauze was >10 minutes in 5 of 7 wounds (mean hemostasis time, 9.11 ± 0.59 minutes). Extraterrestrial regolith increased the time spent clotted compared with pain gauze, with CSM-MGS-1C and CSM-LHT-1 remaining clotted for 3.25 ± 1.33 minutes (*P* = .43; *n* = 6) and 3.38 ± 0.96 minutes (*P* = .24; *n* = 6), respectively, within 10 minutes. The mean hemostasis times were 6.75 ± 1.33 minutes and 6.62 ± 0.96 minutes, respectively, with 2 of 6 wounds achieving hemostasis >10 minutes for CSM-MGS-1C and none of the wounds taking >10 minutes to reach hemostasis for CSM-LHT-1. The NWA-869 chondrite meteorite significantly increased the time spent clotted to 5.58 ± 0.84 minutes within 10 minutes compared with plain gauze (*P* < .01; *n* = 6). The mean hemostasis time was 4.42 ± 0.84 minutes, and none of the wounds took >10 minutes to reach hemostasis with NWA-869.

Blood loss followed a similar trend (Figure 4C). Plain gauze had 69.5 ± 25.5 g of blood loss, whereas CSM-MGS-1C and CSM-LHT-1 had blood loss of 35.2 ± 11.7 g (*P* = .97) and 27.4 ± 6.1 g (*P* = .77), respectively, which were encouraging but not statistically significant, given the number of replicates used in this pilot study. Wounds treated with the NWA-869 meteorite had significantly less blood loss (14.0 ± 8.4 g; *P* < .05) than those treated with plain gauze.

## 4 Discussion

Regolith simulants were used to assess the potential of extraterrestrial regolith to activate clotting, which is a first step toward evaluating its ability to safely control hemorrhage from trauma in humans. While there have been efforts to use human serum albumin as a binder to produce “extraterrestrial regolith biocomposites” [27], this is, to our knowledge, the first effort demonstrating the use of regolith as a medical device. Although other terrestrial silicate-based materials are already used extensively for hemorrhage control on Earth [9], extraterrestrial silicates are distinct due to their formation under anhydrous and inorganic conditions, leading to distinct chemical and mineralogical compositions and physical properties. Our study clarifies the hemostatic potential of silicates found in extraterrestrial environments, which is more relevant to space-based trauma care.

ETRS from the Moon and Mars activated clotting in a FXII-dependent manner, as tested in 3 complementary assays. In the clotting turbidity assay, ETRS significantly reduced the clotting time in a FXII-dependent manner. All 4 ETRS behaved similarly to kaolinite, with the high-clay Martian simulant CSM-MGS-1C performing slightly better than the Lunar simulants and CSM-MGS-1.

Similarly, the thrombin generation assay showed trends comparable to those of the turbidity assay. All 4 regolith simulants significantly reduced the time to thrombin generation, as did kaolinite. In this assay, instead of using FXII-depleted plasma, FXIIa was directly inhibited with CTI, which was also used in the chromogenic assay for FXIIa activity in plasma. The chromogenic assay demonstrated the direct effects of the simulants on FXIIa-like activity. All the simulants led to cleavage of the FXIIa substrate, though CSM-MGS-1C and kaolinite were the most potent. Together, these results demonstrate that regolith simulants have procoagulant effects, which are mediated by the activation of FXII to FXIIa.

The mineral silicate components of the ETRS were also assessed individually for their hemostatic potential in this study. The silicate minerals can be roughly grouped into a framework and chain structure group consisting of plagioclase, pyroxene, and olivine; a phyllosilicate group consisting of Na-montmorillonite, Ca-montmorillonite, and vermiculite; and a fibrous silicate subgroup consisting of palygorskite and serpentine. The phyllosilicates generally showed greater hemostatic potential in the fibrin clot formation and thrombin generation assays. The FXIIa chromogenic assay also showed that the phyllosilicates activated FXII upon the addition of pyroxene. Interestingly, Ca-montmorillonite was the only phyllosilicate to have a modest activating effect, although it had strong hemostatic potential in the fibrin clot formation and thrombin generation assays. The hemostatic potential of the phyllosilicates is likely due to their higher surface area and, consequently, higher surface negative charge density. The prevalence of phyllosilicates in Martian regolith supports their use for hemorrhage control in Martian environments.

Unexpectedly, in both the fibrin clot formation assay and the thrombin generation assay, many of the samples, particularly CSM-MGS-1C and the phyllosilicates, accelerated clotting and thrombin generation in both FXII-immunodepleted and FXIIa-inhibited plasma with CTI. This activation could be attributed to residual FXII in the immunodepleted plasma samples. Additionally, the thrombin generation observed in FXIIa-inhibited plasma suggests that silicates may directly activate other coagulation factors. These results highlight the potency of extraterrestrial regolith as a clotting activator. Further studies are necessary to determine the precise mechanisms underlying these interactions and to confirm the role of silicates in coagulation beyond FXII activation.

The results from the porcine liver puncture model demonstrate that the procoagulant effects observed *in vitro* also apply *in vivo*. Application of the NWA-869 meteorite significantly increased the time wounds remained clotted and decreased blood loss compared with plain gauze. Although the Martian and Lunar simulants did not achieve statistical significance—like the NWA-869 chondritic meteorite compared with plain gauze—both showed evidence of effective hemostasis and reduced blood loss. Nearly all wounds (22/24) achieved hemostasis when an ETRS was applied, whereas most wounds treated with plain gauze alone did not achieve hemostasis. The high variability in blood loss in the control group indicates that our study was underpowered to detect statistically significant differences with the less potent simulants, which is a limitation of this porcine pilot study.

Overall, the results demonstrate the potential role of the *in situ* preparation of hemostatic materials in space. The ETRS behaved similarly to kaolinite, a well-known hemostatic material on Earth. Although not all materials tested were equally hemostatic, we expect that preexisting mineralogical mapping data from landing sites and other areas can predict hemostatic ability based on the types of silicate and content present. The use of regolith for hemorrhage control will likely require some processing steps prior to use due to the possibility of toxic effects or abrasion during use; however, many concerns can be addressed through published ISRU research, which can be integrated into existing mission plans [23,28,29]. On Earth, soils require sterilization to prevent bacteria from being introduced into body cavities and wounds. However, this is not a concern, given that no pathogens have been found on the Moon, Mars, or asteroids, and sterilization will not be required. Nonetheless, Lunar dust is highly abrasive [29] and can be made suitable for use with mechanical tumbling or grinding to produce a powder similar to that used in this study. Powdered regolith would be preferred because its greater surface area would initiate clotting more strongly. Perchlorates in Martian regolith would need to be removed prior to medical use due to their known health effects, including thyroid toxicity [29]. If a long-term base is established, perchlorate remediation is expected to be an integral part of water purification or soil processing systems, which could be readily adapted for medical applications.

For potential usage, regolith could be impregnated onto cotton or polyester gauze from Earth; in more urgent emergency surgical settings, it can simply be poured onto wounds and held in place with plain gauze, clothing, or other fabric after suit removal within a pressurized habitat or vehicle. Following Tactical Combat Casualty Care guidelines, both gauze and granules are likely to have higher efficacy in stopping hemorrhage if followed by at least 3 minutes of direct compression [6,30]. As the scope of emergency surgeries possible on the Moon and Mars has yet to be delineated [31], the use of regolith-based granules or gauzes may play a role in surgeries involving the extremities and abdomen if resources are limited.

As a pilot study, our primary aim was to evaluate the potential of using ETRS for hemorrhage control in large-animal models, which will help guide the development of future larger-scale studies. This study was not intended to suggest replacing current medical supplies, but rather to demonstrate proof of concept that regolith could potentially augment trauma care when locally available resources are required, such as in multiple-casualty scenarios or when resupply is constrained. As a result, this study has several limitations. First, the study was not powered to detect statistically significant differences among all regolith groups. Future studies will include testing beyond the limited pilot study presented here, with sufficient power to detect statistically significant differences in survival. This may also include evaluating long-term toxicity, immunogenicity, and potential mechanical tissue damage caused by the application of processed regolith, which were beyond the scope of this pilot investigation. Second, high-fidelity simulants were used rather than real extraterrestrial regolith. These simulants seek to reproduce the surface mineralogy of the Moon and Mars; thus, it is reasonable to assume that the findings in this study could be extended to the actual regolith of these extraterrestrial bodies [13,32]. However, further studies are required to determine the *in vivo* efficacy of actual extraterrestrial regolith in halting hemorrhage, as it differs in several physical properties due to erosion caused by solar winds and micrometeorite strikes [33]. Furthermore, terrestrial models of hemorrhage do not fully replicate the unique physiological and environmental conditions of space, including microgravity and radiation exposure, which alter coagulation dynamics [[34], [35], [36], [37]]. Finally, it would be valuable to assess the efficacy of a Martian regolith simulant representative of the regional landscape in which landings and a base would be built. Given that this regional regolith is likely to be high in phyllosilicates [38], a strong hemostatic effect would be expected.

In summary, extraterrestrial regolith may potentially serve as a practical hemostatic material for future long-term space missions. Although current research limits the use of extraterrestrial regolith to life-threatening situations when no other options are available, the *in situ* production of medical equipment could lessen the burden on payload constraints and future resupply missions. These results further highlight the potency of soil-based materials in hemostasis, supporting that soil silicates are a coagulation FXV in the coagulation cascade [9].
